# Involvement of the *pagR* gene of pXO2 in anthrax pathogenesis

**DOI:** 10.1038/srep28827

**Published:** 2016-07-01

**Authors:** Xudong Liang, Enmin Zhang, Huijuan Zhang, Jianchun Wei, Wei Li, Jin Zhu, Bingxiang Wang, Shulin Dong

**Affiliations:** 1 National Institute for Communicable Disease Control and Prevention, Chinese Center for Disease Control and Prevention, State Key Laboratory for Infectious Disease Prevention and Control, 102206, Beijing, China; 2 Huadong Medical Institute of Biotechniques, 210002, Nanjing, China; 3 Lanzhou Institute of Biological Products Co. Ltd, 730046, Lanzhou, China

## Abstract

Anthrax is a disease caused by *Bacillus anthracis*. Specifically, the anthrax toxins and capsules encoded by the pXO1 and pXO2 plasmids, respectively, are the major virulence factors. We previously reported that the pXO1 plasmid was retained in the attenuated strain of *B. anthracis* vaccine strains even after subculturing at high temperatures. In the present study, we reinvestigate the attenuation mechanism of Pasteur II. Sequencing of pXO1 and pXO2 from Pasteur II strain revealed mutations in these plasmids as compared to the reference sequences. Two deletions on these plasmids, one each on pXO1 and pXO2, were confirmed to be unique to the Pasteur II strain as compared to the wild-type strains. Gene replacement with homologous recombination revealed that the mutation in the promoter region of the *pagR* gene on pXO2, but not the mutation on pXO1, contributes to lethal levels of toxin production. This result was further confirmed by RT-PCR, western blot, and animal toxicity assays. Taken together, our results signify that the attenuation of the Pasteur II vaccine strain is caused by a mutation in the *pagR* gene on its pXO2 plasmid. Moreover, these data suggest that pXO2 plasmid encoded proteins are involved in the virulence of *B. anthracis*.

Anthrax is a zoonotic disease caused by the spore-forming, gram-positive bacteria, *Bacillus anthracis*. This pathogenic strain was isolated by Robert Koch in 1876. The concept of immunization for anthrax using a suitable vaccine was first investigated in 1880, when Louis Pasteur developed and tested the first live, attenuated anthrax vaccine in sheep using a subculture of *B. anthracis* that had been grown at a high temperature to obtain the attenuated strain for immunization. Max Sterne followed by developing a live anthrax spore vaccine for use in animals in 1939, thus establishing the practice of preventing the occurrence of infectious disease using attenuated pathogens as vaccines^1^,^2^.

The major virulence factors of *B. anthracis* are encoded by genes on two large plasmids. Mikesell *et al*. first reported the discovery of a *B. anthracis* plasmid with a molecular weight of 110 MDa, naming it PBA1 3. It was later demonstrated that the strains developed by Pasteur for his vaccine, Pasteur I and Pasteur II, had both lost the plasmid PBA1 after attenuation. This revealed a likely attenuation mechanism of Pasteur’s vaccine strains^3^. Thereafter, Green and Uchida both reported the discovery of the *B. anthracis* capsule plasmid, pXO2 4,5. In order to unify the plasmid names, PBA1 was renamed pXO1. The main pathogenic factors of anthrax, the toxins and capsule, are encoded by pXO1 and pXO2 plasmid, respectively. Uchida cloned the pXO1-encoded transcriptional regulation protein gene *atxA* (anthrax toxin activator)^6^, and identified the increase in carbon dioxide (CO_2_) and bicarbonate (HCO_3_^−^) that occurs upon translocation of the pathogen from the external environment to the host^7^,^8^. *atxA* is located on the pXO1 pathogenicity island, and McKenzie *et al*. identified a total of 15 pXO1-encoded genes and three chromosomal genes that are regulated either separately or synergistically by *atxA* and CO_2_ 9. AtxA can initiate the expression of *cya* that codes for edema factor (EF), *lef* that codes for lethal factor (LF), and *pagA* that encodes for the protective antigen (PA) protein, by binding to the respective promoters^10^,^11^. Hoffmaster and Koehler demonstrated that the anthrax toxin activator gene *atxA* is also associated with CO_2_-enhanced, non-toxin-associated gene expression in *B. anthracis*^12^. AtxA controls the expression of the pXO2-harbored capsule operon by indirect control of the *acpA* and *acpB* regulators on the pXO2 plasmid^13^,^14^,^15^. *pagA* has since been described as the first gene of a bicistronic operon, of which the second gene, *pagR*, encodes a weak auto-repressor. A 300 bp gene located downstream of *pagA* on pXO1, *pagR,* represses the expression of *pagA* and *atxA.* PagR also controls expression of certain CO_2_/*atxA*-activated transcriptional fusions on pXO1 that do not correspond to toxin genes. Regulation of these fusions, *pagA*, and *pagR* may be due to changes in *atxA* levels. The involvement of *pagA* or *pagR* in the transcription of the S-layer genes was therefore tested, and it was demonstrated that PagR binding could activate *eag* transcription either by blocking a less efficient upstream *eag* promoter or, non-exclusively, by directly activating the more efficient second promoter. Therefore, *pagR* has been defined as an intermediate effector between *atxA* and the genes it controls, at least in the case of both S-layer genes. It is also noteworthy that *pagR* transcription is controlled, or more precisely, activated, by *atxA*^16^,^17^,^18^.

We recently identified that three attenuated vaccine strains obtained through high-temperature subculture, including Pasteur II, retained the pXO1 plasmid^19^. In order to determine whether other factors also contributed to the attenuation, we sequenced the pXO1 and pXO2 plasmids and created gene replacement strains wherein the mutated pXO1 and pXO2 genes were restored. Our results reveal that the specific mutation on the pXO1 plasmid may not act to attenuate the Pasteur II vaccine strain, whereas the pXO2 mutation affects the *pagR* gene, which may facilitate positive regulation of anthrax toxin expression.

## Results

### pXO2 in Pasteur II displays high sequence identity with the Ames ancestor strain

**Figure 1:**
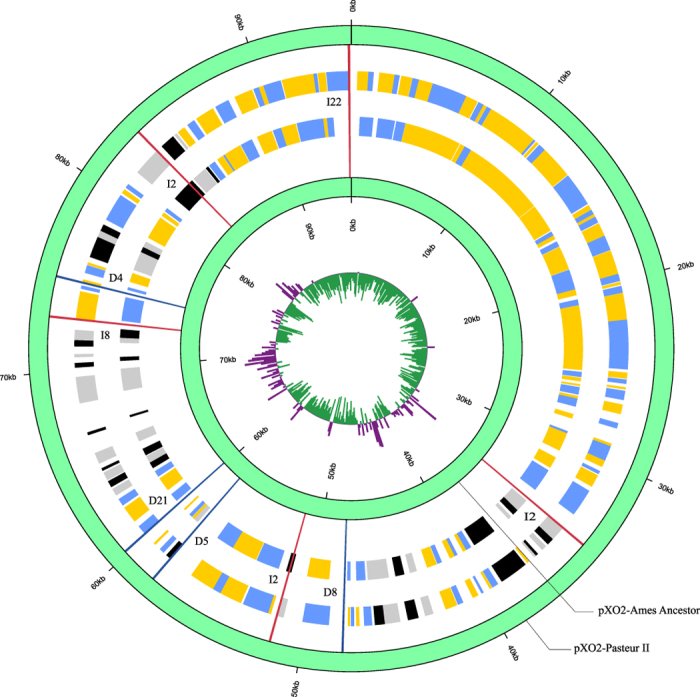
Plasmid map of pXO2 from Pasteur II and Ames. Genome structure and location displayed with GC skew. The order of the rings from the inside of the graphic out is as follows: pXO2-Ames, pXO2 Pasteur II. Yellow and blue represents ORFs on the sense strand in pXO2-Pasteur II, black and gray ORFs on blue the antisense strand in pXO2-Pasteur II. On the GC-skew ring, purple represents GC-skew plus, green signifies GC-skew minus. Orange lines indicate an indel signified by I for insertion and blue lines signified by D for deletion, followed by the number of inserted or deleted nucleotides. The predicted genes on pXO2 of Pasteur II have similar functions with those of pXO2 in Ames.

We predicted the pXO2 plasmid would contain 210 genes. Nearly 90% of the predicted genes were between 100 to 2000 bp. Genes were classified into 12 COG categories similar to Ames Ancestor strain (data not shown). The pXO2 plasmid of Pasteur II and the pXO2 plasmid of the Ames strains display 99% sequence identity. Co-linearity analysis showed gene deletions or insertions at nine sites; no homologs of *pagA* gene was found on pXO2 plasmid (Fig. 1). These results were submitted to the NCBI and the sequence submission data was confirmed under the ID: BankIt1886077 pXO2_Plasmid KU525621. Altogether, we found 16 total mutations sites, including the seven mutations on the pXO1 plasmid we uncovered previously (Supplementary table 1)^19^.

### Identification of specific mutations on pXO1 and pXO2 in the Pasteur II strain

**Figure 2:**
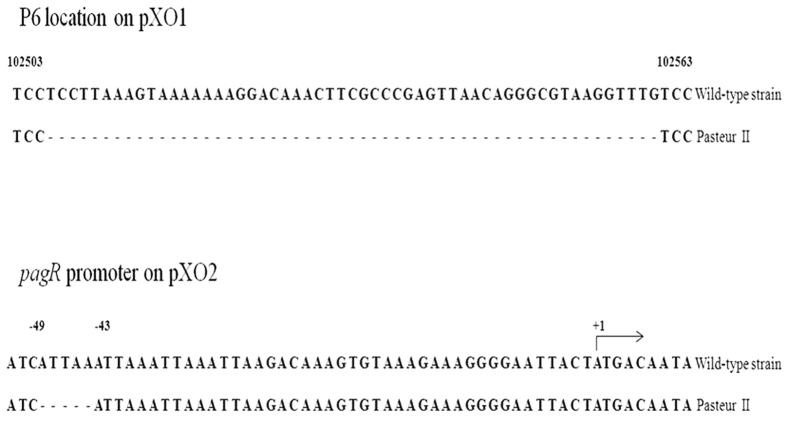
The two deletions of interest on the pXO1 and pXO2 plasmids of the Pasteur II strain. The upper panel shows the 55 bp deletion on pXO1, the lower shows the 5 bp deletion in the *pagR* promoter region on pXO2.

To delineate the variations in the 16 mutations on the pXO1 and pXO2 plasmids in Pasteur II, we amplified and sequenced the corresponding plasmids using 100 wild-type *B. anthracis* strains preserved in our laboratory. Results from the sequencing showed that there were two deletions, one each on the pXO1 and pXO2 plasmids that occurred only in the Pasteur II strain; these two mutations sites were not present on the plasmids from any of the other 100 strains. The specific mutations were a 55 bp deletion (102615–102669, named p6) on the pXO1 plasmid and 5 bp deletion (58519–58523) on the pXO2 plasmid (Fig. 2). Interestingly, the 5 bp deletion is in the promoter region of the *pagR* gene on pXO2 and silences the expression of the *pagR* gene on pXO1; *pagR* on pXO2 is homologous to *pagR* on pXO1, displaying 70% amino acid sequence identity with the *pagR* gene found on the pXO1 plasmid.

### The 5 bp deletion on the promoter region of the *pagR* gene on pXO2 silences *pagR* expression resulting in decreased expression of protective antigen (PA) on pXO1

**Figure 3:**
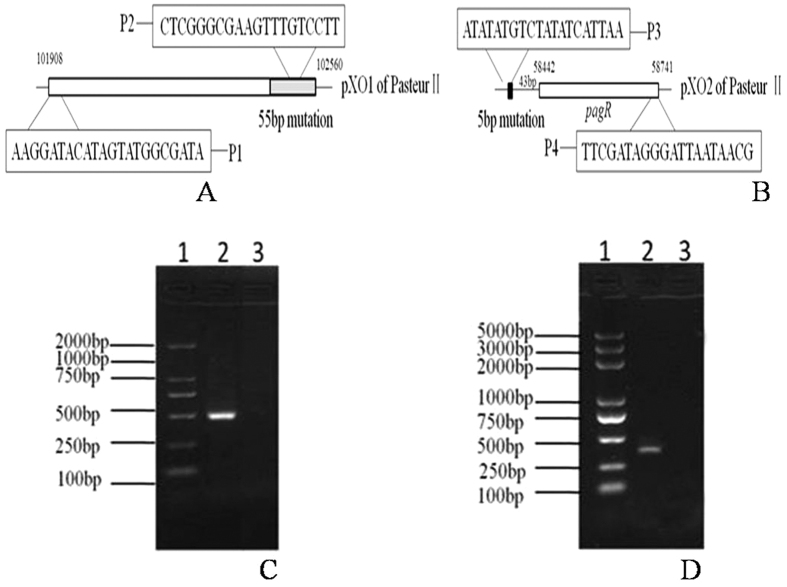
PCR identification of the complementation strains Pasteur III (3C) and Pasteur IV (3D). PCR assay was used to confirm the complemented strain. (**A,B)** are graphical representations of the amplified regions of the Pasteur II plasmid, with the location of the 55 bp deletion on pXO1 represented by the gray box and the 5 bp deletion on pXO2 represented by the black box. Oligonucleotides and their locations are indicated as follows in (**A**,**B)**: P1 and P2 were used for 55 bp complementation confirmation (Pasteur III), and P3 and P4 for 5 bp complementation confirmation (Pasteur IV). (**C)** Lane 1, DNA ladder; Lane 2, PCR product of complemented strain Pasteur III. Lane 3, absence of PCR amplification in Pasteur II. (**D)** Lane 1, DNA ladder; Lane 2, PCR product of complemented strain Pasteur IV; Lane 3, absence of PCR amplification in Pasteur II.

To test the roles of these mutations on Pasteur II attenuation (deletions of 55 bp on pXO1 and 5 bp on pXO2), we constructed the Pasteur III and Pasteur IV strains in which the deletions on pXO1 and pXO2 are replaced by wild-type *B. anthracis* strains sequences using homologous recombination. We designed specific primer sets to confirm the complemented strains via PCR, which harbored wild-type *B. anthracis* and mutation site sequences to determine the success of our gene complementation (Fig. 3). One primer was universal, the other either located in the 55 bp mutation site (P2 primer, Fig. 3A) or whose 3′ end matched the 5 bp mutation site (P3 primer, Fig. 3B). The DNA sequence could thereby be amplified from the gene replacement strain, but not from the original Pasteur II strain (Fig. 3C,D). Gene complementation was further verified by sequencing.

**Figure 4:**
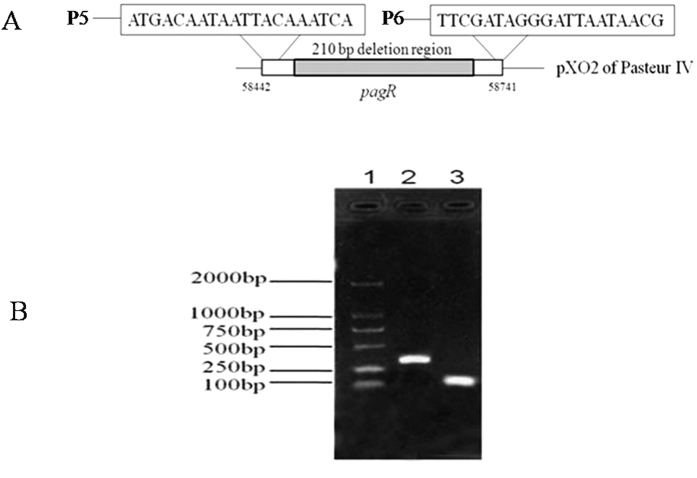
Deletion of the *pagR* gene as identified by PCR amplification in Pasteur IV strain. (**A)** Graphical representation of *pagR* on pXO2. Gray box represents the 5 bp deletion region in the *pagR* promoter. Primers P5 and P4 were used for PCR amplification. (**B)** Lane 1, DNA ladder. Lane 2, PCR product of Pasteur IV; Lane 3, PCR product of Pasteur V.

Based upon sequence analysis, there are *pagR* homologs on both the pXO1 and pXO2 plasmids (named *pagR-XO1* and *pagR-XO2*, respectively). Considering that the 5 bp (58519–58523) deletion on pXO2 is located −43 bp to −49 bp upstream, in the promoter region of *pagR-XO2*, it may affect expression of this gene. Using transcription start site prediction, we deduced that the transcription start site of *pagR-XO2* likely initiates at a region upstream of the 5 bp site; as such, we hypothesize that the 5 bp deletion may result in silencing the expression of *pagR-XO2*, which influences the expression of PA proteins on pXO1. To test our hypothesis we constructed a *pagR-XO2* gene deletion strain Pasteur V, which was also confirmed by PCR (Fig. 4) and sequence analysis.

**Figure 5:**
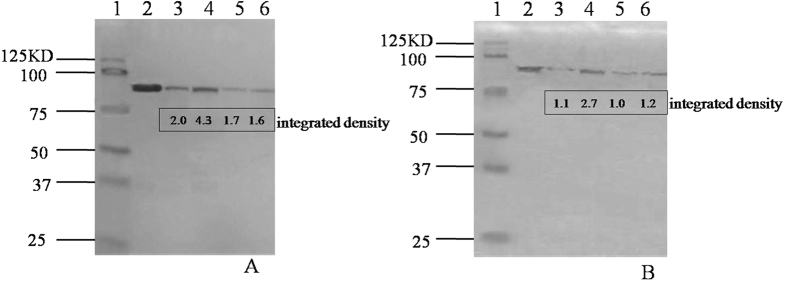
Western blot detection of PA (A) and LF (B) expressed in variant strains. The integrated density of the protein bands was measured using ImageJ software. (**A**) PA was detected by hybridization with anti-PA antibody. Lane 1, control PA (200 ng); Lane 2, Pasteur II; Lane 3, Pasteur IV; Lane 4, Pasteur III; Lane 5, Pasteur V. (**B**) LF was detected by hybridization with anti-LF antibody. Lane 1, control LF (20 ng); Lane 2, Pasteur II; Lane 3, Pasteur IV; Lane 4, Pasteur III; Lane 5, Pasteur V.

A western blot assay showed that the expression level of the PA protein in Pasteur III (Pasteur III is Pasteur II complemented with a wild-type version of the 55 bp mutation region of pXO1) was not significantly different from that in the Pasteur II strain, whereas in the Pasteur IV strain, PA expression increased significantly (Fig. 5A). These results suggest that the attenuation of the Pasteur II strain is related to the missing component of the *pagR* gene promoter on the pXO2 plasmid, and not the unique mutation on the pXO1 plasmid. The deletion in the *pagR* gene promoter on the pXO2 plasmid appears to decrease the expression of the PA protein, which confers a loss of the transporter for LF, whose cytotoxic activities require that they be translocated via the PA protein (Fig. 5B). This indicates that, as a result of this cascade, the *pagR* gene promoter deletion on pXO2 leads to the attenuation of the Pasteur II strain. This is also a possible mechanism for the attenuation of the veterinary Sterne vaccine strain, which has lost its pXO2 plasmid entirely. In addition, the PA protein was weakly detected in Pasteur II by western blot (Fig. 5A), demonstrating that the Pasteur II strain produces a small amount of PA, which may induce antibodies against PA in vaccinated animals. This level of PA production in the Pasteur II strain likely explains why the Pasteur II strain showed both perfect protection and residual toxicity.

We extracted RNA from Pasteur II, Pasteur III, Pasteur IV, and Pasteur V, and quantified transcription levels of key genes using qPCR. Results showed that the mutation in the *pagR* gene promoter reduced transcription of the *pagR* gene on pXO2 (Table 1). This assay also showed that transcription levels of *pagA, lef, pagR-XO2, SlayA* and *atxA* were eight times higher in Pasteur IV than in the original Pasteur II strain, indicating that the *pagR-XO2* gene encoded by pXO2 positively regulates virulence genes and promotes protein expression. However, determining whether this regulation is direct or indirect will require further investigation. Expression levels of *pagA, lef, cya, pagR-XO2,* and *atxA* in the Pasteur V strain showed no significant change when compared to those in the attenuated Pasteur II strain.

### *pagR* contributes to the virulence of Pasteur II in an animal model

**Figure 6:**
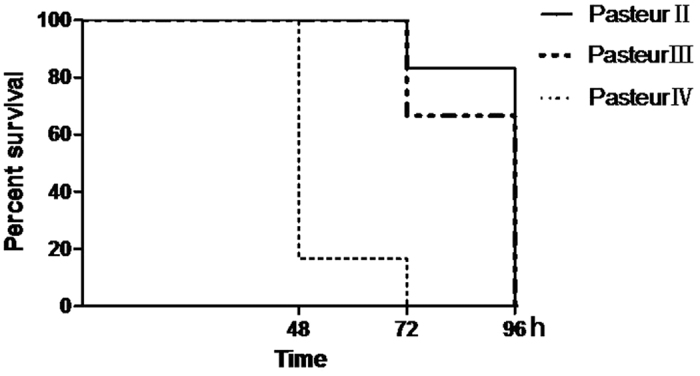
Survival times of BALB/c mice infected with *B. anthracis.* Six mice each were infected with either Pasteur II (solid line), Pasteur III (hashed line), or Pasteur IV (dotted line) vaccine strains of *B. anthracis.* Percent of surviving mice shown as a function of time in hours PI.

Eighteen BALB/c mice were injected subcutaneously with 1 × 10^4^ CFU of Pasteur II, Pasteur III, or Pasteur IV in 0.2 ml, and survival status was observed at 24 h time points post-infection (PI). Of the six mice injected with Pasteur IV, all died within 72 h PI; five died by 48 h PI. Of those injected with Pasteur II, one of the 6 died at 72 h PI and all had died by 96 h PI. In the group infected with Pasteur III, two of the 6 died at 72 h and the rest by 96 h PI (Fig. 6). These results indicate that the Pasteur IV strain might be more virulent than Pasteur II, and that partial attenuation of the Pasteur II vaccine strain may occur.

## Discussion

Almost all anthrax vaccines in use or in development rely on the PA protein as the primary immunogen. The first reported attenuated anthrax vaccine strains, Pasteur I and Pasteur II, enjoyed wide acceptance for the immunization of livestock in Europe and other countries after an immunologic trial thereof was successfully conducted by Louis Pasteur in 1880 20. Use of the live Pasteur strains for vaccination was later discontinued because of residual virulence, to be replaced by the Sterne vaccine strain on account of its reduced toxicity and increased immunogenicity in 1939 21. Sterne-like strains (pXO1^+^pXO2^−^) are routinely used to vaccinate animals against anthrax, and in some countries, such as Russia and China, these strains are also used in humans. In other countries (e.g., the US and the UK), only cell-free vaccines, containing anthrax toxin PA protein components, are licensed for use in humans^22^. Recently, enhancements of the immune response through the addition of cofactors that target Toll-like receptor (TLR) signaling have been reported as well^23^, and several of these, including those containing mutant PA variants with less toxicity and higher immunogenicity, are currently in development^24^,^25^,^26^.

Our previous research showed that the Pasteur II attenuated *B. anthracis* vaccine strain still carried the pXO1 plasmid even after being subcultured at high temperatures^19^. We speculate that this attenuation was caused by mutations of other genes that occurred as a result of the high temperature subculture. To test this hypothesis, we amplified and sequenced the pXO1 and pXO2 plasmids using overlapping PCR primers followed by comparison with the plasmid sequences of the Ames strain. After screening against 100 wild-type strains deposited in our laboratory, we identified two specific mutations sites present only in the Pasteur II vaccine strain. One is a 55 bp deletion on the pXO1 plasmid located at an intergenic region. The other, on the pXO2 plasmid, is a 5 bp deletion in the promoter region of the *pagR-XO2* gene that abrogated expression of this gene.

It has been reported that the *pagR* gene on pXO1 participated in the negative regulation of the *pagA* gene, also on pXO1. The *pagR-XO2* gene on pXO2 has 70% amino acid sequence homology with *pagR-XO1* on pXO1, yet no research illustrating a potential function has been reported until now.

Homologous recombination was used to replace the deleted nucleotides on pXO1 and pXO2 with wild-type sequences, and a *pagR-XO2* replacement strain, Pasteur IV, was also constructed. The RT-PCR assay indicated that the *pagR-XO2* gene on pXO2 was involved in the regulation of expression of the *pagA* and *lef* genes by negatively regulating transcription of *pagR-XO1* on pXO1. This suggests that the mutual coordination and restraint between *pagR* genes on the pXO1 and pXO2 plasmids act to determine and balance toxin gene expression, as demonstrated both in the western blotting and animal model experiments.

The role *pagR-XO2* plays in reducing transcription of *pagA* on pXO1 of the Pasteur II vaccine strain is supported by the performance of the *pagR-XO2* deletion strain Pasteur V. The Sterne vaccine strain and other strains not containing the pXO2 plasmid (with its *pagR-XO2* gene) all display reduced toxin expression resulting in vaccine attenuation. However, the mechanism that underlies the relationship and interference between *pagR-XO1* and *pagR-XO2* will need further exploration.

We have demonstrated that the attenuation of the *B. anthracis* vaccine is related to the participation of the *pagR-XO2* gene on the pXO2 plasmid in the regulation of toxin gene expression on pXO1, indicating a close link between the two plasmids constituting a functional network. Changes in other toxicity-associated genes on the bacterial chromosome may also contribute to virulence reduction in this bacteria. Lethality results in the mouse models indicate that the Pasteur II and Pasteur IV strains are only marginally attenuated and certainly too lethal to qualify as potential live vaccines suitable for use in animals or humans. However, these experiments clarify the genetic background of the currently available Pasteur II vaccine strain and will help efforts to maintain its stability during vaccine manufacture, including with regards to its production of virulence factors.

## Methods

### Pasteur II vaccine strain

The Pasteur II vaccine strain was donated by the Institute of Lanzhou Biological Products. The strain was originally generated at the Pasteur Institute in France in 1934 and was introduced into the Institute of Lanzhou Biological Products in 1935. The Pasteur II strains were preserved in freeze-dried milk at the institute in 1972. The vaccine strain used in this experiment was recovered in LB medium and was verified using traditional biochemical tests in 2011.

### Overlap PCR and sequence assembling

The overlap PCR primers were designed and synthesized as previously described^19^. Briefly, sequences of pXO2 plasmids from the Ames ancestor strain were used as reference sequences for designing of primers. Fragments averaging 3000–4000 bps in length with 300–400 bp overlaps between any two adjacent fragments were used as PCR amplicons. PCR primer sequences are listed in Table 2. Genomic DNA of *B. anthracis* strain Pasteur II was used as the template. TransStart^®^ FastPfu DNA Polymerase (Transgen Co.), was used in all the reactions. PCR products were purified and sequenced, and the sequences were aligned and assembled using Mega 5.0 software. To minimize the possibility of PCR or sequencing errors corrupting our results, primers were designed by which to perform PCR and DNA sequencing to confirm each instance of nucleotide deletion or insertion that was detected via comparison to the Ames strain. Sequences of pXO2 in *B. anthracis* vaccine Pasteur II were inserted into plasmids *in silico* and genes were identified with Glimmer 3.02 software and annotated according to the KEGG and Swiss-Prot databases. Finally, Pasteur II pXO2 mutations were compared to homologous pXO2 plasmids from 100 *B. anthracis* wild-type strains to locate genetic mutations specific to the Pasteur II *B. anthracis* vaccine strain by PCR. This protocol was repeated for the pXO1 plasmid^19^.

### Construction of P6 gene replacement strain in pXO1

Two primers were designed: 101908upF/*Bam*: 5′GCTCGGATCCAAGGATACATAGTATGGCGATA3′ and 103196dnR/*Bgl*: 5′CGGGAGATCTTTCTATAGTCCTTAAGGTTGGC3′, according to the 55 bp sequence located at position 102615–102669 on plasmid pXO1, which was designated P6 and included the 500 bp both upstream and downstream from the target site. A *BamH*I site was introduced into the upstream primer and a *Bgl*II site was created in the downstream primer. A fragment of about 1000 bp length was harvested by PCR using the wild-type strain as a template. The ligated products of the PCR amplicon and pMAD were both double digested with *BamH*I and *Bgl*II and then transformed into *E. coli*. The resulting recombinant plasmid P6-pMAD was transformed into Pasteur II via electroporation using SCS110 cells. The bacteria containing P6-pMAD integrated onto pXO1 was harvested at 42 °C and then subcultured at low temperatures to screen for erythromycin-sensitive colonies. This gene-replacement strain was verified using PCR and designated Pasteur III.

### Construction of the *pagR* gene replacement strain in the pXO2 plasmid

The same methods used above were applied to replace the 5 bp from position 58519–58523 on pXO2. The designated primers used were *pagR-BamHI* 5′GGAGGATCCCTAGGTATCGAATTAATTAAATG3′, and *pagR-BglII*: 5′AGAAGATCTTTATTCGATAGGGATTAATAACG 3′. This replacement strain was designated Pasteur IV.

### Construction of *pagR* gene deletion Pasteur IV strain

The same methods were applied to replace the *pagR* gene. We designed and synthesized primers to a region upstream of the *pagR* gene (*pagR*KOu*p*_*FBam* 5′GGAGGATCCAATAATTGGTGCATACACTTTA3′, *pagR*KOup*-*Rcrossover 5′AATTTTTTCAACCTTGTACTCTATTTCATGATTTGTA3′) and introduced a *BamH*I endonuclease recognition site into the primer. An 800  bp upstream homology arm was amplified by regular PCR using wild-type DNA as template. Downstream primers (*pagR*KOdn*-*Fcrossover 5′CATGAAATAGAGTACAAGGTTGAAAAAATTATTGCGT3′ and *pagR*KOdn-R*Bgl* 5′AGAAGATCTCTTTATTCCTCAACTATGTAG3′) were also synthesized and a *Bgl*II cutting site was introduced therein. An 800 bp downstream homology arm was also amplified. Since portions of the of *pagR*KOup*-*Rcrossover and *pagR*KOdn*-*Fcrossover sequences are complementary to each other, a mixture of upstream and downstream regions was used as the template of the resultant PCR amplification wherein *pagR*KOup*-*F*Bam* and *pagR*KOdn*-*R*Bgl* were used as primers. The resultant PCR products digested with *BamH*I and *Bgl*II were cloned into the pMAD plasmid, which was then digested using the same enzymes to produce the recombinant *pagR*-pMAD plasmid. As before, this recombinant plasmid was activated using SCS110 cells and electroporated into the Pasteur IV strain. The target gene deletion was selected from the erythromycin sensitive strains and confirmed with PCR. This *pagR* gene deletion strain was designated as Pasteur V.

### Western blotting analysis

Strains were cultured overnight and culture supernatant was collected and filtered through 0.22 μm pore syringe filters. Five mL of supernatant was mixed with an equal volume of 95% ethanol and incubated at 4 °C overnight followed by centrifugation. The precipitate was allowed to dry and then resuspended in 50 μl PBS. Following the addition of loading buffer, the sample was heated to 100 °C for 5 min. Twenty μl of each sample (total protein content 5 μg) was loaded onto a 12% SDS-PAGE gel. Following electrophoresis, samples were transferred onto a nitrocellulose membrane (Bio-Rad). Membranes were blocked for 1 h in TBS-T (20 mL Tris base, 137 mL NaCl, 0.1% Tween20) containing 5% dry milk at room temperature. Membranes were subsequently incubated with anti-PA antibody or anti-LF antibody (Thermo Scientific) diluted 1:2000 in TBS-T in 5% milk overnight at 4 °C. Membranes were washed in TBS-T and incubated with goat anti-mouse IgG-HRP (horseradish peroxidase) secondary antibody diluted to 1:4000 in TBS-T in 5% milk for 1 hour at room temperature. After once again washing with TBS-T, the proteins were detected using Pierce™ ECL Western Blotting Substrate (Thermo Scientific) according to the manufacturer’s instructions. The expression levels of the proteins were quantified by measuring the signal of the target bands using an ImageJ software. Commercial purified PA and LF (List Biological Laboratories, Inc.) were used as controls in the experiment.

### RNA isolation and quantitative real-time PCR analysis (qPCR)

Overnight cultures of the experimental strains were incubated at 37 °C with shaking in LB medium until growth reached mid-log phase (OD600 = 0.6). Bacterial cells were then harvested by centrifugation, and total RNA was prepared by the RNeasy Mini Kit (Qiagen) according to the manufacturer’s instructions. Specific primers were designed and synthesized according to each target (Table 3). cDNA was synthesized from 1 μg RNA using Superscript^®^ III Reverse Transcriptase (Invitrogen) and randomized hexamer primers according to the manufacturer’s protocol. The cDNA product was diluted fivefold and then amplified by qPCR. The expression levels of genes in different strains were compared according to their CT values. 16S rRNA was used as a reference for normalization.

### Animal infection

The animal infection assay was performed using male BALB/c mice aged 6 to 8 weeks and weighing 18 to 20 g. Mice were randomly divided into 3 groups and injected with 10^4^ CFU of Pasteur II, Pasteur III, or Pasteur IV in 0.2 ml via inner thigh subcutaneous injection. The inoculated mice were observed constantly for the first 60 min, then once every 12 h.

### Ethics statement

All experiments involving animals were performed in accordance with protocols approved by the Animal Care and Use Committee of the National Institute for Communicable Disease Control and Prevention, China CDC, and according to the medical research regulations of the Ministry of Health, China.

## Additional Information

**How to cite this article**: Liang, X. *et al*. Involvement of the *pagR* gene of pXO2 in anthrax pathogenesis. *Sci. Rep.* **6**, 28827; doi: 10.1038/srep28827 (2016).

## Supplementary Material

Supplementary Information

## Figures and Tables

**Table 1:** Comparison of transcript levels by qPCR analysis between mutation strains vs Pasteur II.

Strain	Change in expression level(n-fold)a								
	pag	lef	cya	pagR-XO1	pagR-XO2	abrB	atxA	SlayA	spoOA
Pasteur III vs Pasteur II	0	0	−4	0	0	−8	0	−4	4
Pasteur IV vs Pasteur II	+8	+8	+4	−8	+8	+8	+8	−4	0
Pasteur V vs Pasteur II	0	0	0	0	0	+8	0	−8	−8

+Increased copy number, indicating positive regulation by *pagR* gene.

−Decreased copy number, indicating negative regulation by *pagR* gene.

**Table 2:** Primers used in overlap PCR.

Primer		Sequence(5’-3’)
pXO2-1	for	TTCCTGATTGTGCGATAAGT
	rev	CGCTTGTTAGGTGGTTCTAC
pXO2-2	for	GATTGCATAATATCGGGTAG
	rev	ACAGATCAATTGCGTAGAAG
pXO2-3	for	AATCGCAACTCCTAACATCA
	rev	GCAGATCAGCAGCGCATTAT
pXO2-4	for	AAGAATTGCCAACGATAAGG
	rev	GAATTGCCCGTACAAGTAGG
pXO2-5	for	TCCGTCTCCACTCGCAAACT
	rev	TGTCCCTGAAACGCATGTTA
pXO2-6	for	AAATTAAAGTAACGCATGAT
	rev	GAGAATTTCGCTACGGACTC
pXO2-7	for	GCTCGAATTGCCTTAGTAGA
	rev	ATCAACTTTCCCGACCATGC
pXO2-8	for	TTCGGTGATTGTTGTACTGG
	rev	TGAAGGCGAAAGCGATGTGT
pXO2-9	for	AAATCTCTTATTCGCACTAA
	rev	TATTTAAGAGAATGCCGTAA
pXO2-10	for	CGGAATATTAGCATATAGTA
	rev	ACAGTAATAGTTAGCGGTCT
pXO2-11	for	GTCTGAATCATTTCAACACG
	rev	GCATAGATGCCATAGAAGCA
pXO2-12	for	CTGGGCACAACAATCAGTT
	rev	TAATGGTTGCGGTAAGTGGT
pXO2-13	for	AAGAACGGCGAGATTACTGC
	rev	TTCCAATTACTTGCCCTAAG
pXO2-14	for	TCTGTTTATGAGGGGGAGAC
	rev	ATCGCTTTGTATTCACTCAG
pXO2-15	for	AAAGAGAACGCCTAGAGTGC
	rev	CAGAAACTCCCGATTATGTG
pXO2-16	for	GAGAAGGTTTCCGAATGTTC
	rev	AGTCGCTAGGGCAATCAGTA
pXO2-17	for	TAAGTTGCCGTATGATCTAC
	rev	ACCGATTAAGCGCCGTAAAG
pXO2-18	for	AGAACGCAGGCTTAGATTGG
	rev	TATAGAAGCAGGCGGGATGA
pXO2-19	for	GTATCATGTCTGTTCGAGTC
	rev	GGAACCAACCTTACAAGAGC
pXO2-20	for	ATCGCTTCTTTAATATAGGT
	rev	AAGTTAAATTCAGCGTATCT
pXO2-21	for	ATTGCCCTATTAACGATGCT
	rev	CTTTCTTCATTTGCGGTGTG
pXO2-22	for	ACGTGGAAATCGCTCTTTCA
	rev	CCTGAAGTATCGGTGATACA
pXO2-23	for	TTAACAGATATTCCCGATGA
	rev	GGGCAGTTGCACCGTTCTAT
pXO2-24	for	TTCAACGCAATATACCCTAC
	rev	ACAGGAATTCGTCGATGCTA
pXO2-25	for	GATGGCAAGGCTCTACGAGA
	rev	TTTGAAGAAATTATGCGACC
pXO2-26	for	GCACCGTCCAATATTACATC
	rev	AGAAGGACAGCTGATACCCA
pXO2-27	for	GAATTTAAAGACCGAATCAG
	rev	GAGCGGATTAATCGTACAAC
pXO2-28	for	AATCGAGTCGTGGCGTTCAT
	rev	GAAGAAGGGCTGCATACTTG
pXO2-29	for	TAGCTCCATTCGGCATTAAG
	rev	TTAGATTTGGGTGACGGTAT
pXO2-30	for	CCTTTCATCACCGAACTAAC
	rev	AGAGATATACAACGGGTTAC
pXO2-31	for	ATCCTTATCTTCCCTTTCCG
	rev	TTAAACGAAGAGTAGAGGTG
pXO2-32	for	TTATTATCAAGAGCCTTGGT
	rev	GAAAAGAATTCGAAACAGTC
pXO2-33	for	TTAATGAACCCAATGCATAA
	rev	TTTCGAACGTATACTGTAGA
pXO2-34	for	AGAGGGGGAAAGCACAACTT
	rev	TCACAACACTCCTGGTAATA

**Table 3:** Primers used in real-time RT-PCR.

Target gene		Primer sequence(5’-3’)
16SrRNA	for	CTACAATGGACGGTACAA
	rev	CTACAATCCGAACTGAGAA
slayA	for	AAGGAAGTAGAGGCAGAA
	rev	GCACGACTTATTGGAGAT
pag	for	TACAAGTGCTGGACCTAC
	rev	CCGTATATCCTTCTACCTCTAA
lef	for	TATCCAGCATAATCATCC
	rev	GAACAATATACACATCAAGA
atxA	for	GTAGCGTCTATAACCTCAG
	rev	TTGCTGTCTGTGGTAATAG
cya	for	CCATAGAACGGTATTAGAGT
	rev	TTCAGCACATCAATCCTA
abrB	for	GTTACCGTCAGATACTTC
	rev	GATGAATTAGGTCGTGTAG
spoOA	for	TCTACTGTTGTTGCTGAT
	rev	GAGTCATATTCGTCAAGTG
pagR-XO1 (pagR on pXO1)	for	ATGAACTTTACAAACATAAAGCA
	rev	TATTGGATAGGGTTTAACAACTT
pagR-XO2 (pagR on pXO2)	for	GCTAAACGGAATACAAATAACTA
	rev	GGGATTAATAACGCAATAATTTT
