# Supplementary material for: Involvement of the pagR gene of pXO2 in anthrax pathogenesis
Source: Sci Rep. 2016 Jul 1;6:28827. doi: 10.1038/srep28827 (PMC4929452; doi:10.1038/srep28827)
Supplement: Supplementary Information [file srep28827-s1.doc]

**Involvement of the *pagR* gene of pXO2 in anthrax pathogenesis**

Xudong Liang^1*§^, EnminZhang^1*^, Huijuan Zhang^1^, Jianchun Wei^1^, Wei Li^1^, Jin Zhu^2§^, Bingxiang Wang^3^, Shulin Dong^3^

1 National Institute for Communicable Disease Control and Prevention, Chinese Center for Disease Control and Prevention, State Key Laboratory for Infectious Disease Prevention and Control, 102206, Beijing, China

2 Huadong Medical Institute of Biotechniques, 210002, Nanjing, China

3 Lanzhou Institute of Biological Products Co. Ltd, 730046, Lanzhou, China

*These authors contributed equally to this study.

§Author to whom correspondence should be addressed; E-Mail: liangxudong@icdc.cn, zhujin1968@njmu.edu.cn

Xudong Liang, National Institute for Communicable Disease Control and Prevention, Chinese Center for Disease Control and Prevention, State Key Laboratory for Infectious Disease Prevention and Control, Changbai Road 155, 102206, Changping, Beijing, China. Tel: +86 10 58900771; fax: +86 10 61731691

Jin Zhu, Huadong Medical Institute of Biotechniques, 293 Zhongshan Dong Road, 210002, Nanjing, P.R.China.

**Supplementary** **table 1 Insertion and deletion sites in two plasmids**

| plasmid | Region | Insertion（I）/Delection（D） | Sequence |
| --- | --- | --- | --- |
| pXO1 | 4204-4221 | D18 | TCTGCTGCATAATAAAGG |
|  | 29327-29411 | I85 | ATTCATCTCGATATACTCTAATTCCATCTGTTTCATACCAAGCTTGAAGAATAGCTTCGATCTCTTCTTCAGATATAAGAATCGT |
|  | 48616-48618 | D3 | AAC |
|  | 86532-86543 | I12 | AAGCAGCAAAAG |
|  | 102615-102669 | D55 | TCCTTAAAGTAAAAAAAGGACAAACTTCGCCCGAGTTAACAGGGCGTAAGGTTTG |
|  | 149297-149305 | I9 | TTATTATTA |
|  | 169601-169648 | D48 | TTCCATTCCTTCTGCAAATCCTCTCGCTTGGCTAATTTCATCAAAATA |
|  | 47863-47870 | D8 | ATTTTTAT |
|  | 51535-51536 | I2 | AC |
| pXO2 | 58519-58523 | D5 | ATTAA |
|  | 72752-72759 | I8 | TGTATCTT |
|  | 74787-74790 | D4 | TATA |
|  | 83142-83143 | I2 | AG |
|  | 94752-94773 | I22 | TTCTTCTTCCGTTTTTGTCATT |
|  | 94792-94794 | I3 | GCA |
|  | 94922-94925 | I4 | TGTA |
